# Complete Annotated Genome Sequences of Four *Klebsiella pneumoniae* Phages Isolated from Sewage in Poland

**DOI:** 10.1128/genomeA.00919-17

**Published:** 2017-11-09

**Authors:** Łukasz Labudda, Dominik Strapagiel, Joanna Karczewska-Golec, Piotr Golec

**Affiliations:** a Department of Molecular Biology, Faculty of Biology, University of Gdańsk, Gdańsk, Poland; b Biobank Lab, Department of Molecular Biophysics, Faculty of Biology and Environmental Protection, University of Łódź, Łódź, Poland; c Laboratory of Molecular Biology, Institute of Biochemistry and Biophysics, Polish Academy of Sciences, Warsaw, Poland; d Department of Bacterial Genetics, Institute of Microbiology, Faculty of Biology, University of Warsaw, Warsaw, Poland

## Abstract

Four lytic phages, vB_KpnP_BIS33, vB_KpnP_IL33, and vB_KpnP_PRA33 of the *Podoviridae* family and vB_KpnM_BIS47 of the *Myoviridae* family, which act against animal-pathogenic *Klebsiella pneumoniae* strains, were isolated from sewage plants in Poland. They possess double-stranded DNA genomes of 41,697 bp, 41,335 bp, 40,605 bp, and 147,443 bp, respectively.

## GENOME ANNOUNCEMENT

*Klebsiella pneumoniae* is a facultative anaerobic Gram-negative bacterium that causes a wide range of diseases in humans and in domestic and farm animals. As a pathogen, it can acquire resistance to carbapenems, which are often considered to be drugs of last resort (1). One promising alternative for antibiotic treatment of carbapenem-resistant *K. pneumoniae* infections might be phage therapy. Lytic bacteriophages (phages) and/or their gene products, such as lysins, can easily be used as therapeutic agents against bacteria, as they are host specific and generally show no side effects.

Four lytic *Klebsiella* phages, vB_KpnP_BIS33, vB_KpnP_IL33, vB_KpnP_PRA33, and vB_KpnM_BIS47, were isolated by a standard enrichment method (2) using *K. pneumoniae* strains that had previously been isolated from seals or dogs. Water samples for phage enrichment were collected from sewage plants in Biskupiec (BIS33 and BIS47), Iława (IL33), and Prabuty (PRA33) in Poland. Genomic DNA from the phages was isolated with a modified phenol-chloroform extraction method (3). Sequencing was performed on an Illumina NextSeq500 platform, generating 6 million, 7.1 million, 10 million, and 17.2 million paired-end reads (2 × 150) for PRA33, IL33, BIS47, and BIS33, respectively. Single contigs were obtained for all four genomes by *de novo* assembling the phage sequences using CLC Genomics Workbench version 8.5.1. Annotation was performed using the myRast (4) and UGENE (5) software packages, with auto-annotation and manual editing, respectively. The upstream sequences of all open reading frames were checked for appropriately spaced ribosome-binding sites with homology to GGAGGT. Promoter sequences were identified by the PHIRE program (6), while tRNA sequences were detected with the tRNAscan-SE (7) and ARAGORN (8) software packages. Table 1 shows the summary report for the four sequenced genomes.

**TABLE 1:** Summary report of the *de novo*-assembled *K. pneumoniae* phages from this study

Phage	NCBI BioSample no.	GenBank accession no.	G+C content (%)	Genome size (bp)	No. of CDSs^a^	Avg coverage (×)
vB_KpnP_PRA33	SAMN05892888	KY652723	52.52	40,605	52	>5,000
vB_KpnP_IL33	SAMN05892890	KY652724	52.50	41,335	55	>15,000
vB_KpnP_BIS33	SAMN05892889	KY652725	52.70	41,697	59	>38,000
vB_KpnM_BIS47	SAMN05892891	KY652726	44.62	147,443	267	>5,200

a CDSs, coding sequences.

Based on gene predictions, all four phage genomes contain genes for replication, virion structure, and lysis. Coding sequences (CDSs) were identified also for putative homing endonuclease, helicase, DNA ligase, and DNA polymerase. Additionally, CDSs for terminase, head-tail connector protein, collar protein, putative tail tubular proteins, and tail fiber protein were found. Bacteriophages vB_KpnP_BIS33 and vB_KpnP_IL33 possess their own RNA polymerase, suggesting that they are related to phage T7. The CDSs for holin and therapeutically desired endolysin were detected in all four genomes. Lysogenization genes, such as site-specific integrases and repressors, were not identified in any of the four genomes. Additionally, genome annotation of vB_KpnM_BIS47 revealed 18 tRNA genes.

Whole-genome sequence alignments with BLASTn (9) and molecular phylogenetic analyses with the maximum likelihood method (10) demonstrated that phages vB_KpnP_BIS33, vB_KpnP_IL33, and vB_KpnP_PRA33 are closely related to each other. However, they differ in a few CDSs encoding, e.g., putative endonuclease, protein kinase, or, in most cases, hypothetical proteins. Phages vB_KpnP_IL33 and vB_KpnP_PRA33 represent the same clade as bacteriophage KpV289 (GenBank accession no. LN866626) (11). Phages vB_KpnP_BIS33 and vB_KpnM_BIS47 show more than 93% whole-genome sequence identity with phages KpV763 (accession no. KX591654) and KB57 (accession no. KT934943), respectively.

### Accession number(s).

Nucleotide sequence accession numbers are shown in Table 1.
